# Is Mitochondria Biogenesis and Neuronal Loss Prevention in Rat Hippocampus Promoted by Apigenin?

**DOI:** 10.32598/bcn.10.6.541

**Published:** 2019

**Authors:** Salvatore Chirumbolo

**Affiliations:** 1. Department of Neuroscience, Biomedicine and Movement Sciences, Unit of Human Anatomy, University of Verona, Verona, Italy.

## Highlights

- Apigenin improves spatial working memory in Wistar rats.
- Apigenin promotes mitochondrial biogenesis by activating peroxisome Proliferator-activated receptor Gamma Co-activator 1-alpha (PGC-1α).
- Apigenin prevents neurodegeneration in rat hippocampus.
- The neurodegenerative damages are mediated by mitochondrial biogenesis.
- Apigenin prevents neurodegeneration by inducing PGC-1α.

## Plain Language Summary

In this correspondence, a comment to a recent paper by Nikbakht et al., published in the latest issue of this journal is reported. The flavone apigenin can exert both its antioxidant potential via the usual enzymatic reactive oxygen species scavenging system and the mitochondrial biogenesis via the peroxisome Proliferator-activated receptor Gamma Coactivator 1-alpha (PGC-1α) mitochondrial Transcription Factor A (TFAM) Nuclear Respiratory factor 1 (NRF-1) pathway. The fascinating paper by Nikbakht et al., earns more insightful clues about the activity of apigenin in the prevention of the rat hippocampus neuronal loss caused by the Aβ25-35 injection.

## 1. Introduction

The recent paper by Nikbakht et al., reported that the flavone apigenin (4′,5,7-trihydroxyflavone) improves spatial working memory in adult male Wistar rats (Nikbakht et al., 2019). According to the authors, apigenin induces a protective effect against neurotoxicity caused by the amyloid fragment β-25-35 in a rodent Alzheimer disease model (Nikbakht et al., 2019). According to the authors, besides the antioxidant and anti-apoptotic properties of apigenin that may prevent neurodegenerative damages in the hippocampal area, further mechanisms can be addressed. In this circumstance, it can be suggested that changes in the levels of mitochondrial Transcription Factor A (TFAM), peroxisome Proliferator-activated Receptor Gamma Coactivator 1-alpha (PGC-1α), phospho-adenosine monophosphate (AMP)-activated protein kinase (pAMPK), AMPK, phospho-cAMP-responsive element binding protein (CREB), and nuclear respiratory factor 1 (NRF-1) proteins occur, particularly for the expression of PGC-1α and the phosphorylation of CREB, which is a master tuner of the memory processes (Ashabi et al., 2012).

PGC-1α and its downstream molecules NRF-1 and TFAM increase following a diet enriched in plant derived-flavones. In this circumstance, the mitochondrial biogenesis results in the prevention of hippocampal neuronal loss as a leading mechanism. Flavonoids are mild activators of reactive oxygen species, which should act as signaling molecules and moderate activators of proapoptotic signals with the complex role to tune the cellular survival machinery (Chirumbolo & Bjørklund, 2017). Some pro-apoptotic signals are potent inducers of mitochondrial biogenesis. At the dose used by the authors, 50 mg/kg by oral gavage every day, the flavone apigenin can induce the early expression of procaspase-3, which is crucial for the activation of mitochondrial biogenesis initiators, such as TFAM and NRF-1 (Kim, Ha, Yang, & Son, 2018). In this perspective, a significant role is ruled by the PGC-1α, the expression of which is particularly stringent for mitochondrial biogenesis (Chen, Tao, Li, & Yao, 2018; Niu, Tang, Ren, & Feng, 2018).

Apigenin exerts its antioxidant role also by inducing the activation of PGC-1α, which in turn can attenuate hydrogen peroxide-induced apoptotic cell death by upregulating Nrf-2 via GSK3β inactivation mediated by activated p38 (Choi et al., 2017). This phenomenon would mean that apigenin can exert both its antioxidant potential via the usual enzymatic reactive oxygen species scavenging system and the mitochondrial biogenesis via the PGC-1α/TFAM/NRF-1 pathway. The fascinating paper by

Nikbakht et al., 2019. earns more insightful clues about the activity of apigenin in the prevention of the rat hippocampus neuronal loss caused by the Aβ25-35 injection.

## Ethical Considerations

### Compliance with ethical guidelines

This manuscript contains original and new ideas, which are not submitted for publishing to other publications or published elsewhere. The corresponding and unique author made substantial contributions to the conception or design of the work. No ethical declarations about patients, psychology, and animals are applicable. References are properly cited within the text and in the reference list.
